# COVID-19 and eye care services in Ethiopia

**Published:** 2020-09-01

**Authors:** Esmael Habtamu

**Affiliations:** 1 Chief Executive Director: Eyu-Ethiopia Eye Health Research, Training and Service Centre, Bahirdar, Ethiopia and Post-Doctoral Research Fellow: International Centre for Eye Health, London School of Hygiene & Tropical Medicine, London, UK.

In Ethiopia, COVID-19 is spreading less rapidly than in many other countries, with 831 people infected, 191 recovered, and 7 deaths as of 28 May 2020. However, its impact on every aspect of life has been profound. The government has declared a state of emergency, limiting movement and physical contact to control the spread of infection. The need to prevent infection, but still deliver important health care services, has profoundly challenged the health care sector.

The ministry of health recently released a statement about the need to continue to provide facility-based essential health care services in parallel with the COVID-19 response; these include maternal and child health services, services for communicable diseases such as HIV, TB, leprosy, and malaria, and non-communicable diseases such as severe hypertension, cardiac problems, diabetes mellitus, asthma, and chronic obstructive pulmonary disease. Eye health is not included in this list, and it is not yet certain what will happen with eye care services.

Most eye health units have suspended eye examinations for fear of spreading the SARS-CoV-2 virus, which is responsible for COVID-19. Services are limited to managing injury-related eye emergencies. Elective ocular surgery has been suspended all over the country, and people presenting to eye units with bilateral blindness from cataract, for example, are being turned away, even if surgery would improve vision. There are concerns that the situation is likely to drive people to look for alternative care, such as from traditional healers; this may prove costly for people’s eyesight and general health.

**Figure:**
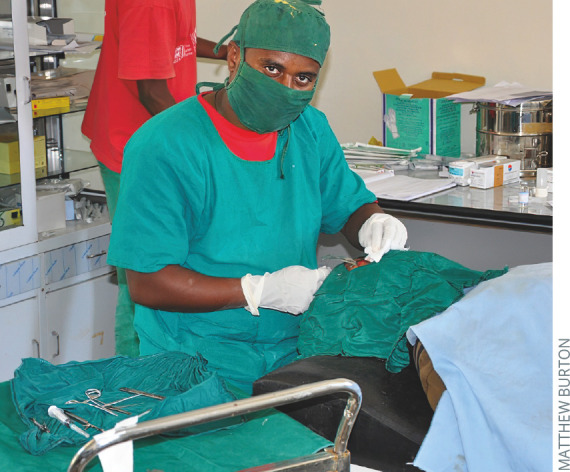
Sight-saving eye surgery has been suspended in Ethiopia due to COVID-19. **ETHIOPIA**

Ethiopia is the country most affected by trachoma. There were intensive trachoma elimination activities throughout Ethiopia before COVID-19. However, following the World Health Organization (WHO) recommendation on neglected tropical diseases (**bit.ly/cov19ntd**), all trachoma elimination activities were suspended, including Zithromax® Mass Drug Administration (MDA) to clear the pool of ocular *Chlamydia trachomatis* infection in the community. This poses a risk that active infection may re-emerge in districts which were on the verge of meeting elimination targets; this compromises several years of collective elimination efforts. Corrective eyelid surgical services to treat trichiasis (the blinding stage of trachoma) have been stopped, leaving hundreds of thousands of people at risk of irreversible vision impairment. Community-based eye health surveys and ongoing research have also been discontinued, leading to considerable delays in the planning and provision of eye health services.

Overall, the COVID-19 situation in Ethiopia is having a profoundly negative effect on the progress made in the last several years as part of the WHO-led VISION 2020 and global trachoma elimination programmes. This has very significant health and socio-economic implications for a country with high rates of poverty and a high burden of blindness from preventable and treatable conditions.

